# Genomic Content of *Bordetella pertussis* Clinical Isolates Circulating in Areas of Intensive Children Vaccination

**DOI:** 10.1371/journal.pone.0002437

**Published:** 2008-06-18

**Authors:** Valérie Bouchez, Valérie Caro, Erwan Levillain, Ghislaine Guigon, Nicole Guiso

**Affiliations:** 1 Institut Pasteur, Unité Prévention et Thérapie Moléculaires des Maladies Humaines, URA-CNRS 3012, Paris, France; 2 Institut Pasteur de Lille, Laboratoire d'Etudes Transcriptomiques et Génomiques Appliquées-Plateforme Biopuces Lille, UMR 8161-IFR 142, Lille, France; 3 Institut Pasteur, Plateforme Santé Publique, Paris, France; Max Planck Institute for Infection Biology, Germany

## Abstract

**Background:**

The objective of the study was to analyse the evolution of *Bordetella pertussis* population and the influence of herd immunity in different areas of the world where newborns and infants are highly vaccinated.

**Methodology:**

The analysis was performed using DNA microarray on 15 isolates, PCR on 111 isolates as well as GS-FLX sequencing technology on 3 isolates and the *B. pertussis* reference strain, Tohama I.

**Principal Findings:**

Our analyses demonstrate that the current circulating isolates are continuing to lose genetic material as compared to isolates circulating during the pre-vaccine era whatever the area of the world considered. The lost genetic material does not seem to be important for virulence. Our study confirms that the use of whole cell vaccines has led to the control of isolates that were similar to vaccine strains. GS-FLX sequencing technology shows that current isolates did not acquire any additional material when compared with vaccine strains or with isolates of the pre-vaccine era and that the sequenced strain Tohama I is not representative of the isolates. Furthermore, this technology allowed us to observe that the number of Insertion Sequence elements contained in the genome of the isolates is temporally increasing or varying between isolates.

**Conclusions:**

*B. pertussis* adaptation to humans is still in progress by losing genetic material *via* Insertion Sequence elements. Furthermore, recent isolates did not acquire any additional material when compared with vaccine strains or with isolates of the pre-vaccine era. Herd immunity, following intensive vaccination of infants and children with whole cell vaccines, has controlled isolates similar to the vaccine strains without modifying significantly the virulence of the isolates. With the replacement of whole cell vaccines by subunit vaccines, containing only few bacterial antigens targeting the virulence of the bacterium, one could hypothesize the circulation of isolates expressing less or modified vaccine antigens.

## Introduction

*Bordetella pertussis*, the agent of whooping cough, is strictly a human pathogen. The incidence of this disease has been markedly reduced in children thanks to high vaccination coverage with vaccines containing inactivated bacterial suspensions, called whole-cell pertussis (Pw) vaccines. Despite the use of efficacious Pw vaccines, the disease still remains a serious public health problem. In fact, with the introduction of childhood vaccination, the circulation of the bacteria has decreased. In absence of natural and vaccine boosters, the immunity of adolescents and adults wanes over time and they become the main source of contamination for infants too young to be vaccinated [1]. The development of subunit vaccines, called acellular pertussis vaccines (Pa), composed of purified detoxified bacterial proteins, allowed the introduction of vaccine boosters for adolescents and adults, now recommended in many developed countries. These boosters are expected to reduce the transmission of the disease to infants and persons at risk, thereby allowing a better control of the disease. However, this may be achievable only if *B. pertussis* isolates are not varying in terms of antigens and especially virulence.

Sequencing of the genome of a representative of each of the three *Bordetella* species pathogenic for humans (*B. pertussis* and *B. parapertussis)* and animals (*B. bronchiseptica)* was at the root of numerous genomic studies and interspecies comparisons [2]. *B. bronchiseptica,* the animal pathogen, is closely related to the common *bronchiseptica*-like ancestor, and two independent events in different *B. bronchiseptica* lineages probably have led to the human-adapted *B. pertussis* and *B. parapertussis* [3]. Differences between *B. pertussis* isolates are mainly due to mutations in structural genes encoding toxins and adhesins, differential expression of genes such as those encoding fimbrial proteins, and gene reduction mostly mediated by IS elements [4]–[7].

Thanks to the availability of clinical isolates from the pre- and post-vaccine era, *B. pertussis* population has been extensively analysed during the last two decades [7]. Bacterial genome typing using multilocus sequences typing (MLST), variable number tandem repeat analyses (VNTR), and pulsed-field gel electrophoresis (PFGE), indicates that genetic diversity is low among the *B. pertussis* population [7]. However, it was possible, by using PFGE and by comparing isolates from different areas of the world, as well as isolates from the pre- and post-vaccine era, to differenciate *B. pertussis* clinical isolates and to classify them in a few (I to VII) PFGE groups [8]-[11]. PFGE groups I, VI and VII are minor groups composed of a few number of isolates. PFGE groups II and III are composed of the Pw vaccine strains and clinical isolates that predominated during the pre-vaccine era but can still be collected, although in minority. PFGE Group V is composed of isolates that predominated in Europe in 1996-1997. Of note, they could already be collected in Europe in the 1960's (Guiso, unpublished). PFGE group IV is composed of isolates predominantly circulating since a decade in areas of the world with a 40-year high Pw vaccine coverage. This last group can be subdivided into different sub-groups according to their PFGE profiles (alpha, beta, gamma), the proportion of which varies by area [9], [12], [13]. We confirmed this PFGE classification by using DNA microarray [4]. Common Regions of Difference (RD) were found in the genome of isolates belonging to the same PFGE group or subgroup: RD1 (from BP0911 to BP0937) and RD2 (from BP1135 to BP1141) were found to be absent in the genome of isolates of most PFGE groups or subgroups but present in the genome of all isolates of PFGE group II, among which one of the French vaccine strain, IM1414. RD3 (from BP1158 to BP1176) was present in the genome of the second French vaccine strain, IM1416, and of PFGE group III clinical isolates collected before 1980. It is deleted, however, from the genome of more recently collected PFGE group III isolates. RD4 (from BP1948 to BP1966) was not present in the genome of PFGE Group IV, subgroup β isolates [4]. Some of these RD were also observed by Brinig *et al.* [5] and by analysis of the temporal evolution of Finnish isolates [6] .

The main objective of the present study was to analyse, by using DNA microarray, whether the loss of genes in *Bordetella* isolates was specific either to a special area of the world or to certain PFGE groups, or whether this represents a general feature of the recent isolates circulating in vaccinated areas of the world. We also checked, thanks to an approach that uses GS-FLX sequencing technology, whether these currently circulating isolates have acquired any additional genetic material, as compared to the sequenced genome.

## Methods

### DNA microarray hybridization

The DNA microarray used, representing 91% of the predicted coding sequences of the sequenced *B. pertussis* reference strain Tohama I, is similar to the one previously described [4]. We have chosen 15 isolates from PFGE group IV, collected between 1982 and 2005. Purified genomic DNA of all isolates was labelled with either Cy3- or Cy5-dCTP, pooled with labelled DNA of the reference Tohama I strain, and hybridized on pre-treated slides. For each sample, a dye-swap experiment was realised simultaneously. After hybridisation, slides were washed with the appropriate buffers and then scanned with the GenePix4000B device.

### Microarray data accession number

Protocols of genomic DNA preparation, purification, labelling, hybridization, as well as raw and normalized microarray data in MIAME format are available at Array Express data repository under accession number E-MEXP-1322.

### PCR validations of microarray results

PCR validations of the DNA microarray results were performed for the RD4 as previously described [4], not only on the DNA from the 15 isolates analysed by DNA microarray but also on DNA from 60 isolates collected in France, 24 in Finland, 3 in The Netherlands, 4 in Buenos Aires (Argentina), 4 in Alger (Algeria), 5 in Cincinnati (USA), 2 in Sweden, 4 in Germany, 5 in Saint-Petersburg (Russia), as well as from 10 isolates of human *B. parapertussis* and 10 of *B. bronchiseptica*. PCR were also run for the RD1 and RD2 on some foreign isolates. A list of the isolates analysed in this study is given in Table S1 (supplemental data). The sequence of the primers for PCR validation is given in Table S2 (supplemental data).

### GS-FLX sequencing

The pyrosequencing of the genome of three isolates (FR0743, FR3080 and FR3713), as compared to the sequenced genome of the reference strain Tohama I, and the analysis of the sequences were carried out by using a 454 GS-FLX NextGen sequencing platform (Roche Diagnostics GmbH, Cogenics Genome Express). Details of the protocols concerning genomic DNA preparation, DNA library preparation, emulsion based-PCR and pyrosequencing on GS-FLX are given in Methods S1.

### Sequence analysis

The sequences produced for each sample were analyzed separately. First, sequences were mapped on the reference genome sequence *Bordetella pertussis* Tohama I, NC_002929, by using the Newbler software V 1.1.02.15. Next, using a Cogenics script, all strictly unmapped sequences were selected and assembled using PHRAP software (version 0.960731, default parameters). Subsequently, the assembled consensus sequences and singletons were mapped on the reference genome of *B. parapertussis* 12822 (NC_002928) and *B. bronchiseptica RB50* (NC_002927) with the SIM2 algorithm [14]. We defined a significant “map” as a sequence alignment with more than 95% of similarity on 95% of the experimental sequence length.

### PCR validations of GS-FLX results

PCR validations of the results obtained thanks to the GS-FLX pyrosequencing were performed for the RD11 to RD14 as previously described (Caro V, Bouchez V, Guiso N, 2008. Is the sequenced *Bordetella pertussis* strain Tohama 1 representative of the species. In revision). A list of the isolates analysed in this study and the primers used for PCR validation is respectively given in Table S1 and Table S2 (supplemental data).

## Results

**Figure 1:**
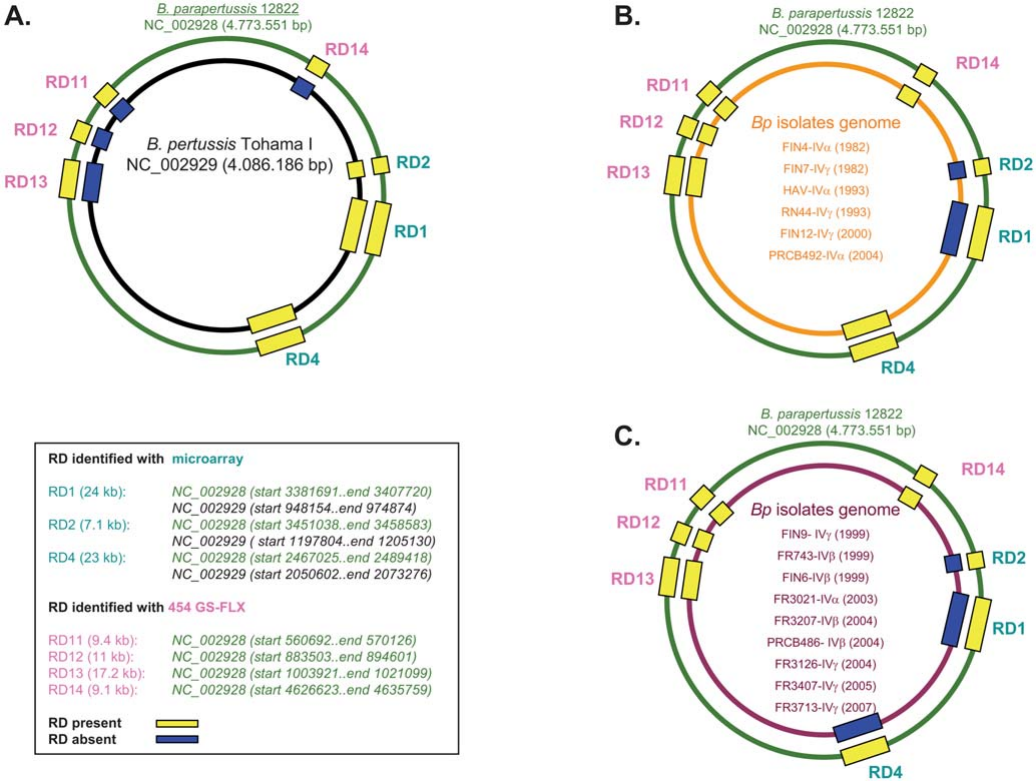
Genomic representation of different RD on B. parapertussis and B. pertussis sequenced reference strains and on B. pertussis isolates RD identified with microarray (RD1, RD2 and RD4) and with GS-FLX (RD11, RD12, RD13 and RD14) were colored according to their presence (yellow) and their absence (blue) on the genome of different B. pertussis isolates. Identified RD size is proportional to their respective length and 10-fold increased in order to better visualize the regions on the genomes. RDs are located according to their position on the B. parapertussis 12822 genome. (A). Circular representation of the genomes of B. pertussis Tohama I (black internal thick line) and of B. parapertussis 12822 (green external thick line) (B). Circular representation of the genomes of B. parapertussis 12822 and of B. pertussis isolates analyzed by microarray and harbouring RD4 (orange internal thick line) (C). Circular representation of the genomes of B. parapertussis 12822 and of B. pertussis isolates analyzed by microarray and not harbouring RD4 (purple internal thick line).

Genomic representation of different RD identified by microarray for French and Finnish isolates are summarized in Figure 1. They indicate that the genomic DNA from all tested isolates of the PFGE group IV, whatever the subgroup, collected between 1982 and 2007 for French isolates and from 1982 to 2004 for Finnish ones, do not possess RD1 and RD2, as compared with the genomic DNA of the reference Tohama I. These RD, (RD1 from BP0911 to BP0937 and RD2 from BP1135 to BP1141), are identical to those observed previously [4], [6]. However, the present observations also indicate that RD4 (from BP1948 to BP1966), which is present in 5 out of 8 genomes of isolates collected before 2000, is deleted in almost all isolates collected after 2001, whatever the subgroup (Figure 1). PCR validations were performed on 5 different coding sequences within RD4. DNA microarray results were confirmed and extended on 60 additional French isolates and on 24 Finnish ones (Figure S1 of the supplemental data). Our observations indicate that the loss of genetic material is continuing. Indeed, significantly more than 78% of PFGE group IV isolates collected since 2001 have lost RD4 *vs* 32% of isolates collected before 2000 (Chi2, p = 8,21326E-06). The loss of RD4 is not specific to these isolates of the PFGE group IV (Figure S1 of the supplemental data), since RD4 is also absent in the DNA of one French isolate of PFGE group III (collected in 2005), in the genomic DNA of three French isolates of PFGE Group VI (collected in 1995), and in the genomic DNA of 2 Finnish isolates collected in 2004 and belonging to the PFGE group VII. All these isolates originated from two countries, France and Finland, where children were highly vaccinated.

Therefore, we tested through PCR validations the presence or absence of RD4 in the genome of a few recent isolates collected from other highly vaccinated areas that we previously analysed by PFGE: 3 isolates from The Netherlands, 4 from Buenos Aires (Argentina), 4 from Alger (Algeria), 5 from Cincinnati (USA), 5 from Saint-Petersburg (Russia), 2 from Sweden and 4 from Germany. As shown in Figure S1 (supplemental data), RD4 was found to be deleted in the genome of almost all isolates recently collected, whatever the area of collection. Our results indicate that RD4 deletion is not specific to any PFGE group or subgroup, and that it does not represent a specificity of recent French or Finnish isolates. The absence of RD1 and RD2 was also confirmed on isolates of different origins (Figure S1 of the supplemental data).

Lastly, we screened for the presence of RD4 in the genome of 10 *B. parapertussis* and 10 *B. bronchiseptica* isolates (old and recent ones). RD4 was present in all genomes tested, suggesting that this deletion is specific to *B. pertussis* isolates recently circulating (Figure S1 of the supplemental data).

As a complementary approach, we used the 454GS-FLX sequencing system. One sequencing run was done simultaneously for the reference strain Tohama I and for 3 French isolates of the PFGE group IV: FR0743 (1999), FR3080 (2004) and FR3713 (2007). The latter isolate was chosen since it was collected from a non vaccinated newborn who died from pertussis. It can be considered to be representative of recent circulating isolates. Run qualification, assembly and mapping characteristics data are presented in Table S3 of supplemental data. For each genome analysed, positive wells represented more than 96% of the total and the more representative length of the sequence obtained was between 240 and 250 nucleotides. Quality filters were applied on the data to eliminate bad quality or usefulness sequences. Passed filter wells for each genome was relatively homogeneous and represented from 20.1Mb for FR3713 to 28.5 Mb for FR743, which corresponds approximately to a fivefold recovery when considering a genome size of 4 Mb for *B. pertussis*. Concerning GS *de novo* assembly characteristics data for contigs greater than 500 bases, results are homogeneous for the 4 isolates, with an average of 1500 contigs each. After mapping the *B. pertussis* Tohama I reference strain, we can observe 44 non-mapping sequences (representing approximatively 2 kb) for the control BPSM, *vs* more than 1000 (representing approximatively 50 kb) for the 3 clinical isolates tested. Those sequences were assembled and mapped to the two reference genomes of *B. parapertussis* and *B. bronchiseptica*. Around 45 kb separated in 4 regions (Figure 1) were found to match *B. parapertussis* and *B. bronchiseptica* sequenced genome (RD11 from BPP0529 to BPP0536, RD12 from BPP0822 to BPP0827, RD13 from BPP0930 to BPP0949 and RD14 from BPP4293 to BPP4301). The four regions were identified and PCR validations were done as previously defined (Caro V, Bouchez V, Guiso N, 2008. Is the sequenced *Bordetella pertussis* strain Tohama 1 representative of the species. In revision) (primers used are detailed in Table S2 and results are presented in Figure S1 of the supplemental data). PCR validations revealed that the 4 regions representing 45 kb were present in the genome of both French vaccine strains, IM1414 and IM1416, in the genomes of isolates collected in the 60's and of presently circulating isolates. Absence of this region seems to be a specificity of the sequenced *B. pertussis* genome. These results confirmed data obtained previously using subtractive hybridization (Caro V, Bouchez V, Guiso N, 2008. Is the sequenced *Bordetella pertussis* strain Tohama 1 representative of the species. In revision). Furthermore, analysis and comparison of the sequence data from the three isolates and from the reference Tohama I strain revealed that the percentages of sequences harbouring repeated structures found within genomes (such as insertion sequence, IS*481*, but the nature of the repeated sequences could not be verified) was increasing temporally. In fact, this percentage is 2.2 for the Tohama I strain but 3.1 for the isolate collected in 1999, 3.6 for the isolate collected in 2004 and 3.9 for the isolate collected in 2007.

## Discussion

Recent bacterial pathogenomics studies revealed unexpected features of bacterial evolution. There are 3 main forces that shaped bacterial genome dynamics evolution: gene gain, gene loss and gene changes [15]. Genomic studies on the *Bordetella* genus, as the sequencing of the three major species pathogens for mammals, showed that *B. pertussis* and *B. parapertussis* are independently derived, host-restricted species that evolved from a *B. bronchiseptica-like* ancestor which can infect many mammalian hosts but also can live independently in the environment [2], [3]. The evolution of the three species occurred mainly by gene loss and gene expression changes. The genome of the derived species, *pertussis* and *parapertussis*, has a smaller size than their ancestor with a considerable increase in the number of IS elements, pseudogenes and many chromosomal rearrangements more particularly for *B. pertussis*. It was also recently shown that allelic polymorphism at homopolymeric tracts is common within the *B. pertussis* genome [16]. This polymorphism could play a role in the adaptation of *B. pertussis* to the Human host but could also play a role for evasion of the immune system. In fact, *B. pertussis* is a vaccine preventable disease. Since now more than 50 years a Pw vaccine is used in some areas of the world with a high coverage for newborns and children. The introduction of vaccination against Pertussis with Pw vaccines has been associated with changes in *B. pertussis* population. Isolates similar to Pw vaccine strains were probably controlled by herd immunity since they are not predominant anymore. The influence of Pw vaccination during several decades on the characteristics of *B. pertussis* population has been previously illustrated [7]. Indeed, we previously observed that when the same vaccine was used during several decades with the same vaccine coverage in two different areas of the world, similar isolates, expressing similar virulence factors, remained circulating [8]. When similar, but not identical (i.e. containing strains expressing different fimbriae), Pw vaccines were used, circulating isolates are very similar but can still be differentiated, for example at the level of fimbriae expression [12]. In many other studies, it was observed that the proportion of isolates corresponding to the type of Pw vaccine strains decreased over time, and that the isolates remaining in circulation were those that exhibited differences to the vaccine strains at the level of protein expression or mutation in structural genes [7]. Furthermore, we previously showed that, in France, the isolates currently circulating also possess less genetic material than the isolates from the pre-vaccine era [4]. Similar observation was recently published in Finland [6]. In the present study, we have shown, using microarray analysis of the genome of 15 isolates and PCR validation of the microarray data on the genome of 111 isolates, that the loss of genetic material is an ongoing process. This loss is neither specific to France nor specific to a particular PFGE group. RD had been previously described between isolates from the pre- and post-vaccine era [4], [6]: RD1 from BP0911 to BP0937 and RD2 from BP1135 to BP1141. We have observed that the majority of isolates circulating since 2001 have also lost RD4 (from BP1948 to BP1966). We previously observed (4) that RD4 deletion was only found in the genome of PFGE group IV*beta* isolates. Our present data show that this deletion is present in the genome of almost all recent isolates tested, whatever the PFGE group (III, IV, VI, VII). The RD4 is composed of 17 ORFs that include 4 pseudogenes and 12 genes encoding proteins involved in: ABC transport and in branched-chain amino-acid transport; a putative ferri-siderophore receptor bfrI; proteins involved in oxidoreduction reactions (oxidoreductase, monooxygenase); a transposase for IS*1663*; and a maleate isomerase. We can assume that these genes are not essential to *B. pertussis* survival inside its human host.

In parallel, using GS-FLX sequencing approach, we have shown that recent isolates did not acquire additional material. We have also confirmed that the sequenced genome is not representative of *B. pertussis* isolates (Caro V, Bouchez V, Guiso N, 2008. Is the sequenced *Bordetella pertussis* strain Tohama 1 representative of the species. In revision) a region of approximately 45 kb was shown to be absent on the Tohama I genome but present in the genome of the 3 tested clinical isolates. This region was still present in the genome of all other clinical isolates of *B. pertussis* but also on the *B. parapertussis* or *B. bronchiseptica* isolates tested, independently of the year of collection. This sequencing approach allowed us to observe that the number of IS elements is temporally increasing or varying between isolates, indicating that the evolution of *B. pertussis* is still in progress.

In our study, we confirm that the use of Pw vaccines has led to the control of the isolates that were similar to the vaccine strains. The predominant circulating isolates are different since they have lost pseudogenes and genes not related to virulence. The loss of genetic material is a dynamic process, not specific to France, but that rather seems to be similar in all areas where Pw vaccines have been used since several decades to immunize children (Alger, Buenos Aires, Finland, France, Saint-Petersbourg). This evolution does not seem to apply to *B. bronchiseptica* and *B. parapertussis*. However, these two species contain much less pseudogenes and insertion sequences than *B. pertussis. B. bronchiseptica* is adapted to many different mammal species but not really to humans and is able to live in the environment. Even if it is sensitive to *B. pertussis*-induced natural or vaccine immunity, this species is not pathogenic for immunocompetent humans [17], [18]. *B. parapertussis*, that evolved from a *bronchiseptica*-like ancestor more recently than *B. pertussis*, might not be submitted to *B. pertussis* immunity [19]–[21] which implies that its evolution is not expected to be similar and therefore needs to be studied.

### Conclusion

Our data confirm that recent *B. pertussis* isolates circulating in areas with high Pw vaccine coverage have less genetic material as compared to isolates from the pre-vaccine era and those circulating some years ago. The herd immunity induced by vaccination has then induced a control of isolates similar to the vaccine strains. Remaining isolates differ from the original ones by loss of genetic material. Loss of genetic material, loss of gene functions are one of the major mechanisms of specialization already observed in many other pathogens, such as *Yersinia, Helicobacter, Salmonella, Pseudomonas* [15], [22], [23]. Huge genome reduction occurred between *B. bronchiseptica* and *B. pertussis* genomes (from 5 to 3.8 Mb) and one may assume that this reduction was simply a response to adapt to its new human host. However, it may also reflect a lack of effective pressure for maintaining these genes over long term evolution [24]. The limited deletions observed in the genome of isolates currently circulating as compared to those circulating during the pre-vaccine era could be the result of modification in the functions involved in host adaptation or a consequence of herd immunity that *B. pertussis* has to face. The lost genetic material does not seem to be important for virulence but could be important for recognition by herd immunity. In fact, some deleted genes encode membrane proteins, which could change the recognition by host cells implicated in immunity. Will these isolates that are losing genetic material not required for their survival in the human host escape herd immunity? This is a major question, especially at a moment where Pw vaccines are replaced by Pa vaccines. Answering this question will necessitate further analyses. However, some observations can already give a piece of answer: Pw vaccines are targeting the whole bacterium and they induce immunity able to control isolates similar to the vaccine strains but not the recent ones. This applies particularly to adolescents and adults whose immunity is waning. By contrast, Pa vaccines are targeting the virulence of *B. pertussis.* Virulence does not seem to have changed over time, both in terms of toxins that are produced and the pathogenicity as evaluated in animal or cellular models [25]. Therefore, it can be hypothesized that all types of isolates will be controlled; especially since the vaccine coverage is increasing thanks to the addition of boosters for adolescents and adults, which is the case in France since 1998, and now also in developed countries. One may argue that the antigens included in Pa vaccines are different from the antigens displayed by the recent circulating isolates [7], especially when considering pertussis toxin and pertactin. However, Pa vaccines were shown to be efficacious in countries where some isolates express different toxin and adhesin [26]. No indication of “escape isolates” is provided from the first epidemiological data in countries such as Sweden, Germany and France that have been using Pa vaccines since only ten years as a replacement of Pw. However, more time may be needed to observe the circulation of such isolates. A question remains whether “escape isolates” are expected to emerge, which does not seem to be necessarily the case if coverage is increasing. Nevertheless, one could expect an increased circulation of isolates expressing less or no vaccine antigens, i.e. less virulent ones or expressing modified isolates. In order to test this hypothesis already proposed [27], surveillance must continue involving standardized and specific diagnoses of the disease as well as analysis and collection of circulating isolates. Such investigations will help to adapt vaccine strategy and are underway in France.

## Supporting Information

Table S1 (0.25 MB DOC)

Table S2 (0.08 MB DOC)

Table S3 (0.05 MB DOC)

Methods S1 (0.04 MB DOC)

Figure S1 PCR validations.
(0.10 MB PDF)
