# Supplementary material for: Genomic Content of Bordetella pertussis Clinical Isolates Circulating in Areas of Intensive Children Vaccination
Source: PLoS One. 2008 Jun 18;3(6):e2437. doi: 10.1371/journal.pone.0002437 (PMC2413009; doi:10.1371/journal.pone.0002437)
Supplement: Table S1 — (0.25 MB DOC) [file pone.0002437.s001.doc]

**Supplementary data**

**Table S1: List of reference strains and isolates used in this study**

*Reference strains or Year of PFGE group Country of origin Reference*

*isolates name collection or subgroup*

***Bordetella pertussis***

BpSM(SM derivated TohamaI) 1954 II France [1]

IM1414 <1954 II France [2]

IM1416 <1959 III France [2]

HAV 1993 IVα France [3]

RN22 1993 IVα France [3]

RN23 1993 IVα France [3]

RN29 1993 IVα France [3]

RN43 1993 IVγ France [3]

RN44 1993 IVγ France [3]

RN50 1993 IVγ France [3]

RN69 1993 IVγ France [3]

RN82 1993 IVα France [3]

RN84 1993 IVα France [3]

FR0056 1993 IVγ France [3]

FR0075 1993 IVγ France [3]

RN127 1994 IVα France [3]

RN136 1994 IVγ France [3]

RN141 1994 IVα France [3]

RN164 1994 IVα France [3]

RN177 1994 IVα France [3]

RN180 1994 IVγ France [3]

FR0096 1994 IVγ France [3]

FR0109 1994 IVγ France [3]

FR0145 1995 III France [2]

FR0146 1995 VI France [3]

FR0182 1995 VI France [3]

FR0184 1995 VI France [3]

FR0282 1996 IVβ France [3]

FR0287 1996 III France [2]

FR0307 1996 III France [2]

FR0371 1997 III France [2]

FR0385 1997 IVβ France [3]

FR0459 1997 IVβ France [3]

FR0620 1998 IVβ France [3]

FR0627 1998 IVβ France [3]

FR0665 1998 IVβ France [3]

FR0668 1998 IVβ France [3]

FR0743 1999 IVβ France [3]

FR0844 1999 IVβ France [3]

FR0854 2000 III France This study

FR1003 2000 IVβ France [3]

FR1062 2000 IVβ France [3]

FR1070 2001 IVβ France [2]

FR1139 2002 IVα France [2]

FR1147 2002 IVα France [3]

FR3015 2003 IVα France This study

FR3020 2003 IVβ France This study

FR3021 2003 IVα France [2]

FR3022 2003 IVβ France [2]

FR3039 2003 IVα France This study

FR3060 2003 III France [2]

FR3079 2004 IVα France This study

FR3080 2004 IVγ France This study

FR3098 2004 III France This study

FR3124 2004 IVα France This study

FR3126 2004 IVγ France This study

FR3127 2004 IVβ France This study

FR3176 2004 IVγ France This study

FR3204 2004 IVγ France This study

FR3207 2004 IVβ France This study

FR3208 2004 IVγ France This study

FR3271 2005 IVβ France This study

FR3279 2005 IVα France This study

FR3342 2005 IVα France This study

FR3348 2005 IVγ France This study

FR3383 2005 III France This study

FR3388 2005 IVβ France This study

FR3406 2005 IVα France This study

FR3407 2005 IVγ France This study

FR3713 2007 IVγ France This study

FIN1 1982 IVα Finland [4]

FIN3 1982 IVα Finland [4]

FIN4 1982 IVα Finland [4]

FIN5 1982 IVα Finland [4]

FIN6 1982 IVα Finland [4]

FIN7 1992 IVγ Finland [4]

FIN8 1992 IVγ Finland [4]

FIN9 1992 IVγ Finland [4]

FIN10 1992 IVγ Finland [4]

FIN11 1992 IVγ Finland [4]

FIN3_PRCB289 1999 IVγ Finland [4]

FIN4_PRCB333 2000 IVγ Finland [4]

FIN5_PRCB301 1999 IVγ Finland [4]

FIN6_PRCB305 1999 IVβ Finland [4]

FIN7_PRCB314 2000 IVγ Finland [4]

FIN9_PRCB291 1999 IVγ Finland [4]

FIN12_PRCB309 2000 IVγ Finland [4]

FIN14_PRCB274 1999 IVα Finland [4]

FIN15_PRCB286 1999 IVα Finland [4]

FIN16_PRCB287 1999 IVα Finland [4]

FIN17_PRCB290 1999 IVγ Finland [4]

FIN18_PRCB292 1999 IVα Finland [4]

FIN19_PRCB293 1999 IVα Finland [4]

FIN20_PRCB296 1999 IVγ Finland [4]

PRCB486 2004 IVβ Finland [4]

PRCB487 2004 VII Finland [4]

PRCB489 2004 IVβ Finland [4]

PRCB490 2004 IVβ Finland [4]

PRCB491 2004 IVβ Finland [4]

PRCB492 2004 IVα Finland [4]

PRCB496 2004 IVβ Finland [4]

PRCB497 2004 IVα Finland [4]

PRCB505 2004 IVα Finland [4]

PRCB513 2004 IVβ Finland [4]

PRCB515 2004 VII Finland [4]

PRCB529 2004 IVβ Finland [4]

S29 2004 IVα Sueden [5]

S41 2004 IVβ Sueden [5]

D34 2005 IVγ Germany [5]

ET501-106 2004 IVα Germany Andre *et al.,* in press

ET501-107 2004 IVβ Germany Andre *et al.,* in press

ET501-108 2004 IVα Germany Andre *et al.,* in press

NL38 2004 IVα The Netherlands [5]

NL40 2004 IVβ The Netherlands [5]

NL44 2004 IVγ The Netherlands [5]

ET501-161 2004 IVβ USA-Cincinatti Andre *et al*., in press

ET501-162 2004 IVγ USA-Cincinatti Andre *et al*., in press

ET501-350 2004 IVβ USA-Cincinatti Andre *et al*., in press

ET501-351 2004 IVα USA-Cincinatti Andre *et al.,* in press

ET501-352 2004 IVβ USA-Cincinatti Andre *et al.,* in press

AR001 2005 IVβ Argentina [6]

AR004 2005 IVβ Argentina [6]

AR006 2005 III Argentina [6]

AR013 2005 IVβ Argentina [6]

DZ002 2003 IVβ Algeria Lazri *et al* , unpublished data

DZ003 2004 IVβ Algeria Lazri *et al* , unpublished data

DZ005 2004 IVβ Algeria Lazri *et al* , unpublished data

DZ007 2004 IVβ Algeria Lazri *et al* , unpublished data

RUS45 1999 IVα Russia-St Petersburg [7]

RUS54 1999 IVβ Russia-St Petersburg [7]

RUS146 2001 IVγ Russia-St Petersburg [7]

RUS205 2004 IVα Russia-St Petersburg This study

Rus200 2004 IVβ Russia-St Petersburg This study

***Bordetella parapertussis***

12822 NA USA [8]

CIP63.2 1960 NA France [9]

Rus115 1960 NA Russia-St Petersburg [7]

CIP107611 1963 NA France This study

CIP107612 1963 NA France This study

BPP010 1998 NA France This study

FR3002 2003 NA France This study

FR3085 2004 NA France This study

FR3222 2004 NA France This study

FR3286 2005 NA France This study

FR3638 2006 NA France This study

FR3676 2007 NA France This study

FR3721 2007 NA France This study

FR3743 2007 NA France This study

***Bordetella bronchiseptica***

RB50 NA USA [8]

DEL 1989 NA France [10]

BB5 NA France [10]

Rem1 1989 NA France [11]

Rem5 1997 NA France [11]

LEG 1999 NA France This study

LORD 1999 NA France This study

LIB 2000 NA France This study

FR3325 2005 NA France This study

FR3417 2005 NA France This study

FR3474 2005 NA France This study

FR3488 2005 NA France This study

FR3525 2006 NA France This study

FR3623 2007 NA France This study

FR3649 2007 NA France This study

FR3718 2007 NA France This study

FR3727 2007 NA France This study
