# Supplementary material for: Genomic Content of Bordetella pertussis Clinical Isolates Circulating in Areas of Intensive Children Vaccination
Source: PLoS One. 2008 Jun 18;3(6):e2437. doi: 10.1371/journal.pone.0002437 (PMC2413009; doi:10.1371/journal.pone.0002437)
Supplement: Table S2 — (0.08 MB DOC) [file pone.0002437.s002.doc]

**Supplementary data**

**Table S2: Primers used for PCR validation**

| *Target Gene^a^* | *Forward Primer (5’-3’)* | *Reverse Primer(5’-3’)* | *Product lenght(bp)* |
| --- | --- | --- | --- |
|  |  |  |  |
| **RD1 validation** |  |  |  |
| BP0911-BP0913 | tcagtcgctggatttcttgg | cgacttctgaaccagcttgc | 2527 |
| BP0924 | atacgagatcggcgtacgag | tagctctcgcgcaacttgac | 897 |
| BP0933-BP0934 | ctcgaaaatgacggtggtg | agggcatggaagatcagttc | 1549 |
|  |  |  |  |
| **RD2 validation** |  |  |  |
| BP1136 | atctgacgcaggatgtgttc | atgtattgctgcaccgaact | 353 |
| BP1141 | gaacgccactgccttctg | cagaacgtgaggagccagat | 254 |
|  |  |  |  |
| **RD3 validation** |  |  |  |
| BP1160 | ccatgctccagttcgaga | gatgaccgacgtcagcttt | 317 |
| BP1166 | tgctcgaccagatcgactac | ttgacgtcctcgaagaacag | 374 |
| BP1176 | gcaccagttcctgggtatg | gtgtggaaggaatgcaggt | 328 |
|  |  |  |  |
| **RD4 validation** |  |  |  |
| BP1948 | gcacaggacaccatcaaga | ggccaggaagaacaccttc | 301 |
| BP1952 | tctacaccaccaacatcacg | gtgcaggatgacctggat | 363 |
| BP1954 | atgggacatgggcaagag | agcaccagtccgggtttt | 399 |
| BP1960 | cgtatcgctacgacaggct | ggaacttgctgtgcacgtag | 324 |
| BP1966 | gctggactacagctttcgc | cactggccgaagtggatg | 320 |
| **RD11 validation** |  |  |  |
| BPP0533 | gtatcgaggcgggcacgatgc | tcggcgggcaggcaagcattc | 268 |
| **RD12 validation** |  |  |  |
| BPP00825 | cagcgacaatacgccagg | gcgtcgtgttaccagggag | 213 |
| **RD13 validation** |  |  |  |
| BPP0944BPP0945 | cgggtctgggcttcgatg | cgcctagggccttgcaac | 196 |
| **RD14 validation** |  |  |  |
| BPP4297 | tccgatggcggtggccag | gtctccgccctggacatc | 301 |
|  |  |  |  |

*^a^*Target gene indicates the gene from which the primer is derived
