# Supplementary material for: Genomic Content of Bordetella pertussis Clinical Isolates Circulating in Areas of Intensive Children Vaccination
Source: PLoS One. 2008 Jun 18;3(6):e2437. doi: 10.1371/journal.pone.0002437 (PMC2413009; doi:10.1371/journal.pone.0002437)
Supplement: Table S3 — (0.05 MB DOC) [file pone.0002437.s003.doc]

**Supplementary data**

**Table S3: Characteristics of the GS-FLX run**

| **GS-FLX Run characterisitics** | **BpSM** | **FR743** | **FR3080** | **FR3713** |
| --- | --- | --- | --- | --- |
| Raw Wells | 166734 | 197317 | 192632 | 178003 |
| Positive Wells (Keypass) | 160528 | 191891 | 187541 | 172701 |
| Dot | 5729 | 6783 | 6344 | 5571 |
| Mixed | 40336 | 36163 | 44292 | 55685 |
| Short Quality | 20085 | 25088 | 24193 | 22671 |
| Short Primer | 134 | 168 | 164 | 124 |
| Passed Filter Wells | 94244 | 123689 | 112548 | 8865 |
| Total Bases Mb | 21,8 | 28,5 | 26,1 | 20,1 |
| **Characterisitics of de novo GS assembly** | **BpSM** | **FR743** | **FR3080** | **FR3713** |
| numberOfContigs | 1664 | 1590 | 1554 | 1545 |
| numberOfBases | 2588393 | 2583520 | 2577860 | 2398544 |
| avgContigSize | 1555 | 1624 | 1658 | 1552 |
| largestContigSize | 12051 | l18810 | 20228 | 15145 |
| Q40PlusBases * | 2419532 | 2481384 | 2460224 | 2253474 |
| **Characteristics of the mapping with the *B pertussis* Tohama I reference strain** | **BpSM** | **FR743** | **FR3080** | **FR3713** |
| numberFullyMapped | 86622 | 111876 | 80005 | 101324 |
| numberPartiallyMap | 173 | 318 | 230 | 265 |
| numberUnmapped | 44 | 1434 | 1016 | 1371 |
| numberRepeat | 7151 | 9708 | 7057 | 9256 |
| numberOfContigs | 2581 | 2548 | 2621 | 2472 |
| numberOfBases | 3397956 | 3253365 | 3171564 | 3243776 |
| **Mapping of unmapped contigs with the *B bronchispetica RB50 reference strain*** | **BpSM** | **FR743** | **FR3080** | **FR3713** |
| **RD 11** | *NA* | BPP0529-BPP0536 | BPP0529-BPP0536 | BPP0529-BPP0536 |
| **RD 12** | *NA* | BPP0822-BPP0827 | BPP0822-BPP0827 | BPP0822-BPP0827 |
| **RD 13** | *NA* | BPP0930-BPP0949 | BPP0930-BPP0949 | BPP0930-BPP0949 |
| **RD 14** | *NA* | BPP4293-BPP4301 | BPP4293-BPP4301 | BPP4293-BPP4301 |

*Dot : sequences giving no signal during the first cycles*

*Mixed : sequences recognized as doubles*

*Short Quality : sequences that do not fit the chosen quality criteria*

*Short Primer : sequences corresponding to the adaptaters used for the library sDNA creation.*

*Q40PlusBases : nb of bases of the contigs with a quality score greater or equal to 40*

*(équivalent score Phrap).*

*NumberFullyMapped : nb of sequences with a complete match*

*NumberPartiallyMapped : nb of sequences with a partial match*

*NumberUnmapped : nb of sequences that do not match*

*NumberRepeat : nb of sequences with multiple significatif matchs*
