# Supplementary material for: Genomic Content of Bordetella pertussis Clinical Isolates Circulating in Areas of Intensive Children Vaccination
Source: PLoS One. 2008 Jun 18;3(6):e2437. doi: 10.1371/journal.pone.0002437 (PMC2413009; doi:10.1371/journal.pone.0002437)
Supplement: Methods S1 — (0.04 MB DOC) [file pone.0002437.s004.doc]

**Methods S1:**

The pyrosequencing of the 4 genomes and the analysis of the sequences were carried out by using a 454 GS-FLX NextGen sequencing platform (Roche Diagnostics GmbH, Cogenics Genome Express).

**Single Strand DNA library**

According to the GS pyrosequencing protocol, gDNA must first be transformed into a library of single-strand template DNA fragments (sstDNA) flanked with amplification and sequencing primer sequences. These sstDNA libraries were prepared using the GS Library Preparation kit (Roche Diagnostics GmbH) according to the manufacturer’s recommendations. Each sample was individually treated. Briefly, 5 μg of gDNA was fragmented by nebulization, which shears double-stranded DNA into fragments ranging from 400 to 800 base pairs, for each of the 4 genomes. Fragment ends were made blunt before the ligation of the adaptors « A » and « B » allowing for both amplification and sequencing. To purify single strand DNA integrating both the A and the B adaptor, the ligation mixture was immobilized onto magnetic streptavidin-coated beads, via the biotin moiety of Adaptor B. After several wash steps to eliminate the unbound fragments without B adaptors, the single strand DNA integrating both the A and B adaptor were melted away from the beads and purified. The quality of the sstDNA was checked by a size range analysis with an RNA 6000 Pico-Assay on the 2100 Bioanalyzer (Agilent Technologies) and the quantification of the sstDNA libaries was carried out by a sensitive fluorescent measurement using the Quant-it™ Ribogreen® RNA assay (Invitrogen).

**emPCR**

Emulsion PCR (emPCR) corresponds to a clonal amplification of the sstDNA library generated previously. For Whole Genome Sequencing application, the emPCR is carried out with the GS emPCR Kit I (Roche Diagnostics GmbH). For each sequenced sample, 8 emulsion amplification reactions were prepared separately.

Briefly, the sstDNA was immobilized onto DNA Capture Beads. The obtained captured DNA library was added to a mixture of amplification mix and oil and vigorously shaken on a Tissue lyser (Qiagen) to create “micro-reactors” containing both amplification mix and a single bead.

Emulsion was dispatched in a 96 well plate and the PCR amplification program according to the manufacturer’s recommendations was performed. After the amplification step, the emulsion was chemically broken, and the beads carrying the amplified DNA library were recovered and washed by filtration. Positive beads were purified by the biotinylated primer “A” that binds to streptavidin-coated magnetic beads. The DNA library beads were then separated from the magnetic beads by melting away the double-stranded amplification products, leaving a population of bead-bound single-stranded template DNA fragments.

Next, the sequencing primer was annealed to the amplified sstDNA. Lastly, the number of beads carrying amplified sstDNA was evaluated with a Z2™ Cell Counter (Beckman Coulter).

**Sequencing run**

The 4 samples were simultaneously sequenced in one GS-FLX run using one 70X75mm Pico-Titer plate device (Roche Diagnostics GmbH) and one GS LR-70 sequencing kit (Roche Diagnostics GmbH). Briefly, the 70X75mm Pico-Titer plate was divided in 4 regions using the Medium Regions Bead Loading Gasket (Roche Diagnostics GmbH). According to the manufacturer’s recommendations, 300 000 DNA beads were loaded per region, followed by the appropriate volume of packing beads and enzyme beads. After the pre-wash run, the sequencing run was launched with the “FullAnalysis” parameter set.

**REFERENCES OF SUPPLEMENTARY DATA**

1. Hot D, Antoine R, Renauld-Mongenie G, Caro V, Hennuy B, et al. (2003) Differential modulation of *Bordetella pertussis* virulence genes as evidenced by DNA microarray analysis. Mol Genet Genomics 269: 475-486. Epub 2003 May 2024.

2. Caro V, Hot D, Guigon G, Hubans C, Arrive M, et al. (2006) Temporal analysis of French *Bordetella pertussis* isolates by comparative whole-genome hybridization *Bordetella pertussis*, Finland and France. Microbes Infect 8: 2228-2235.

3. Weber C, Boursaux-Eude C, Coralie G, Caro V, Guiso N (2001) Polymorphism of *Bordetella pertussis* isolates circulating the last ten years in France, where a single effective whole-cell vaccine has been used for more than thirty years. J Clin Microbiol 39: 4396-4403.

4. Caro V, Elomaa A, Brun D, Mertsola J, He Q, et al. (2006) *Bordetella pertussis*, Finland and France. Emerg Infect Dis 12: 987-989.

5. Hallander H, Advani A, Riffelmann M, von Konig CH, Caro V, et al. (2007) *Bordetella pertussis* strains circulating in Europe in 1999 to 2004 as determined by pulsed-field gel electrophoresis. J Clin Microbiol 45: 3257-3262.

6. Caro V, Bouchez V, Guiso N, Gatti B, Agosti MR, et al. (2007) Pertussis in Argentina and France. Vaccine 25: 4335-4339.

7. Kourova N, Caro V, Weber C, Thiberge S, Chuprinina R, et al. (2003) Comparison of the *Bordetella pertussis* and *Bordetella parapertussis* isolates circulating in Saint Petersburg between 1998 and 2000 with Russian vaccine strains. J Clin Microbiol 41: 3706-3711.

8. Parkhill J, Sebaihia M, Preston A, Murphy LD, Thomson N, et al. (2003) Comparative analysis of the genome sequences of Bordetella pertussis, *Bordetella parapertussis* and *Bordetella bronchiseptica.* Nat Genet 35: 32-40.

9. Khelef N, Danve B, Quentin-Millet MJ, Guiso N (1993) *Bordetella pertussis* and *Bordetella parapertussis:* two immunologically distinct species. Infect Immun 61: 486-490.

10. Gueirard P, Guiso N (1993) Virulence of *Bordetella bronchiseptica*: role of adenylate cyclase-hemolysin. Infect Immun 61: 4072-4078.

11. Gueirard P, Weber C, Le Coustumier A, Guiso N (1995) Human *Bordetella bronchiseptica* infection related to contact with infected animals: persistence of bacteria in host. J Clin Microbiol 33: 2002-2006.
