# Supplementary material for: Genomic Content of Bordetella pertussis Clinical Isolates Circulating in Areas of Intensive Children Vaccination
Source: PLoS One. 2008 Jun 18;3(6):e2437. doi: 10.1371/journal.pone.0002437 (PMC2413009; doi:10.1371/journal.pone.0002437)
Supplement: Figure S1 — PCR validations. (0.10 MB PDF) [file pone.0002437.s005.pdf]

	Gene.ID	Name	S29-2004	S41-2004	D34-2005	NL38-2004	NL40-2004	AR001-2005	AR004-2005	DZ002-2003	DZ003-2004	RUS45-1999	RUS54-1999	Functionnal description
			α	β	γ	α	β	β	β	β	β	α	β	
RD-1	BP0910	transposase												transposase for IS481 element
	BP0911	unknown												putative decarboxylase
	BP0912	unknown												LysR-family transcriptional regulator (Pseudogene)
	BP0913	unknown												putative exported protein
	BP0914	unknown												probable inner membrane component of binding-protein-dependent transport system
	BP0915	unknown												probable inner membrane component of binding-protein-dependent transport system
	BP0916	unknown												putative ATP-binding protein of a transporter (Pseudogene)
	BP0918	unknown												conserved hypothetical protein
	BP0919	gabD												putative succinate-semialdehyde dehydrogenase [NADP+]
	BP0920	unknown												putative exported protein
	BP0921	citB												citrate utilization protein B
	BP0922	unknown												conserved hypothetical protein
	BP0923	unknown												conserved hypothetical protein
	BP0924	unknown												putative transcriptional regulator
	BP0925	unknown												putative fumarylacetoacetate-family hydrolase
	BP0926	unknown												conserved hypothetical protein
	BP0928	unknown												LysR-type transcriptional regulator
	BP0929	unknown												putative membrane protein
	BP0930	unknown												putative CoA ligase
	BP0931	unknown												putative exported protein
	BP0932	unknown												conserved hypothetical protein
	BP0933	unknown												conserved hypothetical protein (Pseudogene)
	BP0934	unknown												hypothetical protein
	BP0938	transposase												transposase for IS481 element
RD-2	BP1134	transposase												transposase for IS481 element
	BP1135	tauD												alpha-ketoglutarate-dependent taurine dioxygenase
	BP1136	fecI (ECF1)												ECF-family sigma factor
	BP1137	fecR												putative signal transduction protein
	BP1138	bfrH												putative ferric siderophore receptor
	BP1139	unknown												putative iron uptake protein
	BP1140	unknown												putative iron uptake protein (Pseudogene)
	BP1141	unknown												putative iron uptake protein
	BP1142	transposase												transposase for IS481 element

PCR validations for RD1 and RD2 for *B. pertussis* foreign isolates

absent

present

PCR validations for RD4 on *B. pertussis* French isolates

Isolate	Type
IM1414<1954(*)	II
BpSM-1954(*)	II
IM1414<1954(*)	II
IM1416<1959(*)	III
IM1416<1959(*)	III
HAV-1993(*)	α
HAV-1993	α
RN22-1993	α
RN23-1993	α
RN29-1993	α
RN43-1993	γ
RN44-1993	γ
RN44-1993	γ
RN50-1993	γ
RN69-1993	γ
RN82-1993	α
RN84-1993	α
FR0056-1993	γ
FR0075-1993	γ
RN127-1994	α
RN141-1994	α
RN164-1994	α
RN177-1994	α
RN136-1994	γ
RN180-1994	γ
FR0096-1994	γ
FR0109-1994	γ
FR145-1995(*)	III
FR145-1995(*)	III
FR146-1995	VI
FR182-1995	VI
FR184-1995	VI
FR282-1996	β
FR287-1996(*)	V
FR287-1996(*)	V
FR307-1996(*)	V
FR371-1997(*)	V
FR385-1997	β
FR459-1997	β
FR620-1998	β
FR627-1998	β
FR665-1998	β
FR668-1998	β
FR743-1999	β
FR743-1999	β
FR844-1999	β
FR854-2000(*)	III
FR1003-2000	β
FR1062-2000	β
FR1070-2001	β
FR1139-2002	α
FR1147-2002	α
FR3015-2003	α
FR3020-2003	β
FR3021-2003	α
FR3021-2003	α
FR3022-2003	β
FR3039-2003	α
FR3060-2003	III
FR3079-2004	α
FR3080-2004	α
FR3098-2004	III
FR3124-2004	α
FR3126-2004	γ
FR3126-2004	γ
FR3127-2004	β
FR3176-2004	γ
FR3204-2004	γ
FR3207-2004	β
FR3207-2004	β
FR3208-2004	γ
FR3271-2005	β
FR3279-2005	α
FR3342-2005	α
FR3348-2005	γ
FR3383-2005	III
FR3388-2005	β
FR3406-2005	α
FR3407-2005	γ
FR3407-2005	γ
FR3713-2007	γ
FR3713-2007	γ

Gene.ID	Name	Functionnal description
BP1947	transposase	transposase for IS481 element
BP1948	unknown ( )	branched-chain amino acid-binding protein
BP1949	unknown ( )	putative permease component of branched-chain amino acid transport system
BP1950	unknown ( )	putative ATP-binding component of branched-chain amino acid ABC transporter
BP1951	unknown ( )	putative ATP-binding component of ABC transporter
BP1952	unknown ( )	putative cytochrome
BP1953	unknown ( )	probable oxidoreductase
BP1954	unknown ( )	putative monooxygenase
BP1959	transposase	transposase for IS1663 element
BP1960	unknown ( )	probable aldehyde dehydrogenase
BP1961	unknown ( )	putative flavocytochrome
BP1962	bfrI (BfrI)	putative ferrisiderophore receptor
BP1963	unknown ( )	putative transcriptional regulator
BP1963BIS	unknown ( )	putative transcriptional regulator
BP1965	unknown ( )	putative exported protein
BP1966	unknown ( )	putative sulfatase

PCR validations for RD4 on *B. pertussis* foreign isolates

Country	Isolate	Type
FINLAND	FIN1-1982	α
	FIN3-1982	α
	FIN4-1982	α
	FIN4-1982	α
	FIN5-1982	α
	FIN6-1982	α
	FIN7-1992	γ
	FIN7-1992	γ
	FIN8-1992	γ
	FIN9-1992	γ
	FIN10-1992	γ
	FIN11-1992	γ
	FIN14_PRCB274-1999	α
	FIN15_PRCB286-1999	α
	FIN16_PRCB287-1999	α
	FIN3_PRCB289-1999	γ
	FIN17_PRCB290-1999	γ
	FIN9_PRCB291-1999	γ
	FIN9_PRCB291-1999	γ
	FIN18_PRCB292-1999	α
	FIN19_PRCB293-1999	α
	FIN20_PRCB296-1999	γ
	FIN5_PRCB301-1999	γ
	FIN6_PRCB305-1999	β
	FIN6_PRCB305-1999	β
	FIN12_PRCB309-2000	γ
	FIN12_PRCB309-2000	γ
	FIN7_PRCB314-2000	γ
	FIN4_PRCB333-2000	γ
	PRCB486-2004	β
	PRCB486-2004	β
	PRCB487-2004	VI
	PRCB489-2004	β
	PRCB490-2004	β
	PRCB491-2004	β
	PRCB492-2004	α
	PRCB492-2004	α
	PRCB496-2004	β
	PRCB497-2004	α
	PRCB505-2004	α
	PRCB513-2004	β
	PRCB515-2004	VI
	PRCB529-2004	β
Sweden	S29-2004	α
	S41-2004	β
Germany	D34-2005	γ
	ET501-106-2004	α
	ET501-107-2004	β
	ET501-108-2004	α
Netherland	NL38-2004	α
	NL40-2004	β
	NL44-2004	γ
USA	ET501-161-2004	β
	ET501-162-2004	γ
	ET501-350-2005	β
	ET501-351-2005	α
	ET501-352-2005	β
Argentina	AR001-2005	β
	AR004-2005	β
	AR006-2005	III
	AR013-2005	β
Algeria	DZ002-2003	β
	DZ003-2004	β
	DZ005-2004	β
	DZ007-2004	β
Russia	RUS45-1999	α
	RUS54-1999	β
	RUS146-2001	γ
	RUS200-2004	β
	RUS205-2004	α

Gene.ID	Name	Functionnal description
BP1947	transposase	transposase for IS481 element
BP1948	unknown ( )	branched-chain amino acid-binding protein
BP1949	unknown ( )	putative permease component of branched-chain amino acid transport system
BP1950	unknown ( )	putative ATP-binding component of branched-chain amino acid ABC transporter
BP1951	unknown ( )	putative ATP-binding component of ABC transporter
BP1952	unknown ( )	putative cytochrome
BP1953	unknown ( )	probable oxidoreductase
BP1954	unknown ( )	putative monooxygenase
BP1959	transposase	transposase for IS1663 element
BP1960	unknown ( )	probable aldehyde dehydrogenase
BP1961	unknown ( )	putative flavocytochrome
BP1962	bfrI (BfrI)	putative ferrisiderophore receptor
BP1963	unknown ( )	putative transcriptional regulator
BP1963BIS	unknown ( )	putative transcriptional regulator
BP1965	unknown ( )	putative exported protein
BP1966	unknown ( )	putative sulfatase

PCR validations for RD4 on *B. parapertussis* and *B. bronchiseptica* isolates

Species	Isolate
BORDETELLA PARAPERTUSSIS	12822
	63.2
	RUS115-1960
	CIP107611-1963
	CIP107612-1963
	BPP010-1998
	FR3002-2003
	FR3085-2004
	FR3222-2004
	FR3286-2005
	FR3638-2006
	FR3676-2007
	FR3721-2007
	FR3743-2007
BORDETELLA BRONCHISEPTICA	RB50
	DEL
	BB5
	REM1-1989
	REM5-1997
	LEG-1999
	LORD-1999
	LIB-2000
	FR3325-2005
	FR3417-2005
	FR3474-2005
	FR3488-2005
	FR3525-2006
	FR3623-2007
	FR3718-2007
	FR3727-2007

Gene.ID	Name	Functionnal description
BP1947	transposase	transposase for IS481 element
BP1948	unknown ( )	branched-chain amino acid-binding protein
BP1949	unknown ( )	putative permease component of branched-chain amino acid transport system
BP1950	unknown ( )	putative ATP-binding component of branched-chain amino acid ABC transporter
BP1951	unknown ( )	putative ATP-binding component of ABC transporter
BP1952	unknown ( )	putative cytochrome
BP1953	unknown ( )	probable oxidoreductase
BP1954	unknown ( )	putative monooxygenase
BP1959	transposase	transposase for IS1663 element
BP1960	unknown ( )	probable aldehyde dehydrogenase
BP1961	unknown ( )	putative flavocytochrome
BP1962	bfrI (BfrI)	putative ferrisiderophore receptor
BP1963	unknown ( )	putative transcriptional regulator
BP1963BIS	unknown ( )	putative transcriptional regulator
BP1965	unknown ( )	putative exported protein
BP1966	unknown ( )	putative sulfatase

PCR validations for RD4

- absent (microarray data)
- present (microarray data)
- absent (PCR validation)
- present (PCR validation)

(*) results from previous study Caro et al., 2006

Validation	Target gene	*B. pertussis* isolates																							*Bpp* isolates					*Bb* isolates		
		Tohamal Clp8132	FIN4-1982	FIN7-1992	HAV-1993	RN22-1993	RN44-1993	FR 0287-1996	FR 0307-1996	FR 0371-1997	FR 0743-1999	FR 0844-1999	FIN6=PRCB305-1999	FIN9=PRCB291-1999	FIN12=PRCB309-2000	FR3021-2003	FR 3080-2004	FR3126-2004	FR3207-2004	PRCB486-2004	PRCB492-2004	FR3279-2005	FR3407-2005	FR 3713-2007	Bpp12822	FR 3002-2003	FR3085-2004	FR 3222-2004	FR 3286-2005	Bb RB50	FR3325-2005	FR3649-2007
**RD11**	**BPP0533**	absent	present	present	present	present	present	present	present	present	present	present	present	present	present	present	present	present	present	present	present	present	present	present	present	present	present	present	present	present	present	present
**RD12**	**BPP0825**	absent	present	present	present	present	present	present	present	present	present	present	present	present	present	present	present	present	present	present	present	present	present	present	present	present	present	present	present	present	present	present
**RD13**	**BPP0944-BPP0945**	absent	present	present	present	present	present	present	present	present	present	present	present	present	present	present	present	present	present	present	present	present	present	present	present	present	present	present	present	present	present	present
**RD14**	**BPP4297**	absent	present	present	present	present	present	present	present	present	present	present	present	present	present	present	present	present	present	present	present	present	present	present	present	present	present	present	present	present	present	present

PCR validations for RD11, RD12, RD13 and RD14

absent

present

isolates used for the run 454 GS-FLX
